# Road Marking Damage Degree Detection Based on Boundary Features Enhanced and Asymmetric Large Field-of-View Contextual Features

**DOI:** 10.3390/jimaging11080259

**Published:** 2025-08-04

**Authors:** Zheng Wang, Ryojun Ikeura, Soichiro Hayakawa, Zhiliang Zhang

**Affiliations:** Faculty of Engineering, Mie University, Tsu 514-8507, Mie Prefecture, Japan; 423de54@m.mie-u.ac.jp (Z.W.);

**Keywords:** markings damage degree detection, boundary feature enhancement, contextual feature information, deep learning

## Abstract

Road markings, as critical components of transportation infrastructure, are crucial for ensuring traffic safety. Accurate quantification of their damage severity is vital for effective maintenance prioritization. However, existing methods are limited to detecting the presence of damage without assessing its extent. To address this limitation, we propose a novel segmentation-based framework for estimating the degree of road marking damage. The method comprises two stages: segmentation of residual pixels from the damaged markings and segmentation of the intact markings region. This dual-segmentation strategy enables precise reconstruction and comparison for severity estimation. To enhance segmentation performance, we proposed two key modules: the Asymmetric Large Field-of-View Contextual (ALFVC) module, which captures rich multi-scale contextual features, and the supervised Boundary Feature Enhancement (BFE) module, which strengthens shape representation and boundary accuracy. The experimental results demonstrate that our method achieved an average segmentation accuracy of 89.44%, outperforming the baseline by 5.86 percentage points. Moreover, the damage quantification achieved a minimum error rate of just 0.22% on the proprietary dataset. The proposed approach was both effective and lightweight, providing valuable support for automated maintenance planning, and significantly improving the efficiency and precision of road marking management.

## 1. Introduction

Road markings are essential elements of road infrastructure that regulate vehicle movement, guide drivers, and provide reliable locational information [1]. They help reduce traffic accidents, ease congestion, and improve road usage efficiency. Moreover, road markings are critical for intelligent driving systems, including lane departure warning, autonomous navigation, and driver assistance [2,3]. In addition, they support robot navigation [4] and assist the visually impaired by providing mobility cues [5]. However, road markings are prone to wear and damage due to weathering, heavy traffic, and aging materials. This deterioration impairs marking visibility and recognition, potentially compromising driving safety and the performance of intelligent systems. In severe cases, it may lead to failures in lane detection or autonomous navigation. Therefore, the timely and accurate detection of severely damaged markings is essential for maintaining traffic safety [6].

Current methods for the detection of road markings mainly fall into two categories: LiDAR scanning and computer vision [7]. LiDAR-based methods offer high accuracy. However, laser sensors have a limited field of view, and their performance is affected by surface material and color. These limitations reduce their suitability for roads with irregular or complex geometries. In contrast, computer vision methods leverage cameras to capture road scenes at various resolutions, providing rich visual cues for detecting various types of road markings. Therefore, this study focuses on computer vision approaches to quantify the degree of damage in road markings.

Machine vision-based road marking detection methods can be broadly divided into two categories: traditional methods based on hand-crafted features [8,9], and deep learning-based image segmentation approaches [10,11,12]. Traditional methods rely on manually designed features tailored to specific processing stages and scenarios. Their reliance on expert knowledge limits effectiveness in complex environments, often resulting in weak feature extraction and unstable detection performance. Moreover, the limited expressiveness of hand-crafted features restricts model generalization and robustness. In contrast, deep learning-based methods demonstrate superior performance in automatic feature representation learning. Among them, segmentation-based approaches are the most widely adopted for road marking detection [13,14,15]. For instance, reference [16] proposes a method based on twin attention modules and maximum stable external regions (MSER) to achieve high-precision road marking segmentation. Building on this, reference [17] employed an autoencoder architecture to aggregate contextual information from sequential images, thereby improving the model’s ability to distinguish lane markings from background regions. In addition to feature fusion for richer context acquisition, increasing the model’s attention to key regions is another effective strategy for enhancing detection performance. Reference [18] proposed a semantic segmentation network incorporating both spatial and channel-wise attention mechanisms, which simultaneously model contextual dependencies and emphasize discriminative features. Moreover, reference [19] Based on the damage rate of road markings, a road marking quality detection network based on YOLOv11 was developed. However, although segmentation-based methods are effective in detecting damaged areas, they often struggle to accurately delineate the complete structure of road markings when the degree of damage is severe. In addition, some studies have attempted to detect damaged road markings using target detection frameworks such as YOLO [20,21]. Despite the high detection speed of such methods, the results usually only determine whether the markings are damaged or not, and it is difficult to further quantify the degree of damage, which limits its application value in fine maintenance. Although deep learning methods have achieved remarkable success in lane markings detection, most existing studies focus primarily on road markings, with limited attention given to other types of road markings and the assessment of their damage severity. Unlike conventional segmentation or detection tasks, damage severity detection requires not only the accurate localization of markings, but also the quantitative evaluation of their deterioration. In real-world scenarios, road markings exhibit diverse types and varying scales, and are often affected by shadows, lighting variations, and occlusions. Moreover, their discontinuous structures and complex shapes pose additional challenges for accurate segmentation.

To address these challenges, this paper proposes a method for quantifying road marking damage by integrating boundary feature enhancement and asymmetric large-field contextual information. The proposed approach consists of two main stages. The first stage segments the residual pixels of the damaged markings to calculate their remaining area. The second stage segments the intact marking region to estimate the total area of the original markings. Accurately reconstructing the complete region of the road markings is crucial for quantifying the degree of damage. However, due to the fine-grained nature of marking boundaries, existing models tend to focus on local features and lack sufficient long-range context. To address this, the second stage incorporates the ALFVC and BFE modules to enhance reconstruction of the complete marking region from damaged inputs.

1. This paper presents a method for detecting the degree of damage to road markings, which consists of region-level segmentation and pixel-level segmentation. The region-level segmentation aims to extract the complete marking area from the damaged markings, while the pixel-level segmentation focuses on identifying the residual marking pixels.
2. The ALFVC module is proposed to acquire rich multi-scale contextual feature information. This module is designed to extract features in both horizontal and vertical directions, thereby capturing the morphological features of the marking with greater accuracy.
3. A supervised BFE is proposed to learn the boundary features (including shape features) of the road markings, thereby enabling the model to accurately segment the complete road markings regions even in blurred, damaged, and occluded areas.

The remainder of this paper is structured as follows. Section 2 introduces the semantic model for road markings segmentation along with related feature enhancement methods and contextual features. Section 3 provides a detailed description of the proposed method. Section 4 presents a comprehensive experimental validation of the proposed method. Section 5 discusses the limitations of the methodology and outlines directions for future research. Finally, Section 6 concludes the paper.

## 2. Related Work

Owing to their strong generalization, adaptability, and high accuracy, deep learning-based methods have been widely applied in road defect detection [22,23,24] and lane line recognition for autonomous driving [25,26,27]. In road scene understanding, the two most widely used approaches are object detection and semantic segmentation. Object detection methods are used to locate road targets and classify them into predefined categories by predicting their bounding boxes and class labels. Studies [28,29] employed conventional object detection approaches to identify damaged road markings with high accuracy. However, it does not quantify the degree of damage to road markings. Semantic segmentation techniques assign a class label to each pixel, enabling the fine-grained delineation of road targets. The work in [30] constructed a comprehensive road marking dataset and used semantic segmentation to successfully distinguish over 15 marking types. In this paper, we propose a novel approach that integrates region-level and pixel-level segmentation within a unified semantic segmentation framework. This method not only enables the accurate segmentation of road markings, but also quantifies their damage severity, supporting timely maintenance decisions for severely deteriorated areas.

### 2.1. Semantic Segmentation Modeling

Semantic segmentation is a crucial task in computer vision that focuses on assigning labels to each pixel in an image, thereby ensuring precise pixel-wise classification. The majority of early semantic segmentation methods were based on the Fully Convolutional Network (FCN) architecture [31], which replaces fully connected layers with convolutional layers to process images of arbitrary size and generate output masks with the same spatial resolution as the input. Building on this foundation, encoder–decoder-based networks, such as U-Net [32], effectively leverage multi-level feature representations. This is achieved through skip connections at symmetric positions, which facilitate the transfer of feature information from the encoder to the decoder. Furthermore, to enhance the resolution of feature maps, the Pyramid Scene Parsing Network (PSPNet) [33] broadens the receptive field of the network by utilizing multiple parallel dilated convolutions. This approach significantly improves the model’s capacity for multi-scale feature extraction. Extending this concept, DeepLabv3 [34] integrates an Atrous Spatial Pyramid Pooling (ASPP) module into the encoder–decoder framework, allowing for feature extraction across various receptive fields through parallel dilated convolutions. However, conventional semantic segmentation methods are limited to local feature extraction and struggle to capture global contextual information, which hinders their performance in accurate boundary delineation. Additionally, over-reliance on local features may lead to overfitting, reducing model generalization and degrading segmentation performance in fine-grained regions. To address this issue, we designed an Asymmetric Large Field-of-View Contextual Feature module and incorporated multi-scale feature extraction to integrate semantic information across different levels.

### 2.2. Contextual Characterization Information

Contextual features are essential for enhancing the performance of semantic segmentation models. Abundant contextual information strengthens the model’s capacity to comprehend and integrate spatial cues, thereby enabling accurate segmentation of occluded or distant objects. Moreover, contextual information provides fine-grained semantic cues, which assist the model in making accurate pixel-level classification. This also helps preserve clear object boundaries in complex scenes. To extract richer contextual features, ref. [34] introduces the Atrous Spatial Pyramid Pooling (ASPP) module to capture multi-level semantic context. The research presented in [35] employs a pyramid network module to integrate contextual feature information at varying scales. The study in [36] utilizes channel attention and spatial attention mechanisms to capture long-range contextual dependencies. Currently, contextual feature extraction methods primarily employ two techniques: larger convolutional kernels and dilated convolutions. Although a larger convolutional kernel can capture a broader range of contextual feature information, it also significantly increases computational complexity. Dilated convolution enlarges the receptive field by inserting “holes” between convolution kernels without increasing parameters; however, these gaps may result in information loss or insufficiency, particularly when handling high-frequency details. Additionally, the discontinuity introduced by dilated convolutions may lead to a “checkerboard effect” or unnatural artifacts in the feature map, adversely affecting the continuity and completeness of feature extraction. To address these challenges, we propose an Asymmetric Large-View Contextual Feature Learning module that captures multi-scale contextual information, aiming to comprehensively enhance the representation of road marking features.

### 2.3. Feature Information Enhancement

Enhancing feature information plays a pivotal role in improving model performance and robustness. The quality of feature representation and expressiveness is optimized through various techniques and methodologies, encompassing the refinement of feature selection and extraction, feature transformation, and the effective fusion of multi-scale and cross-modal features. Enhancing feature information enables models to comprehend and integrate spatial information more broadly, facilitating accurate parsing of complex and diverse data. Studies [37,38] proposed a supervised segmentation approach for glass edge features, effectively achieving comprehensive segmentation of glass. Specifically, the Enhanced Boundary Learning technique in reference [37] makes it possible to segment the edges of glass-like transparent objects more clearly and accurately by refining the boundary features. Meanwhile, reference [38] effectively captures the global information and complex background in the image through Large-Field Contextual Feature Learning, thus improving the robustness and accuracy of glass detection. The research in [39] recognized that features across different layers exhibit varying levels of texture richness. By sequentially fusing features from deep to shallow layers, the expressive power of these features was significantly enhanced. In this paper, the boundary feature information of the labeled line is crucial for segment the original region from the damaged sections. Therefore, we propose a supervised boundary feature enhancement method aimed at learning the fine-grained boundary details of intact markings, thereby enabling more efficient segmentation from the damaged areas.

## 3. Proposed Approach

**Figure 1:**
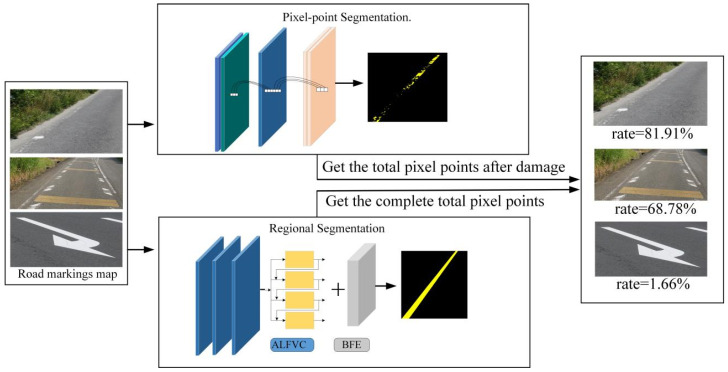
Block diagram of road marking damage detection. The “Pixel-Point Segmentation” stage is used to segment the pixel points left in the damaged road markings. The “Regional Segmentation” stage is used to segment the original region of the damaged marking.

**Figure 2:**
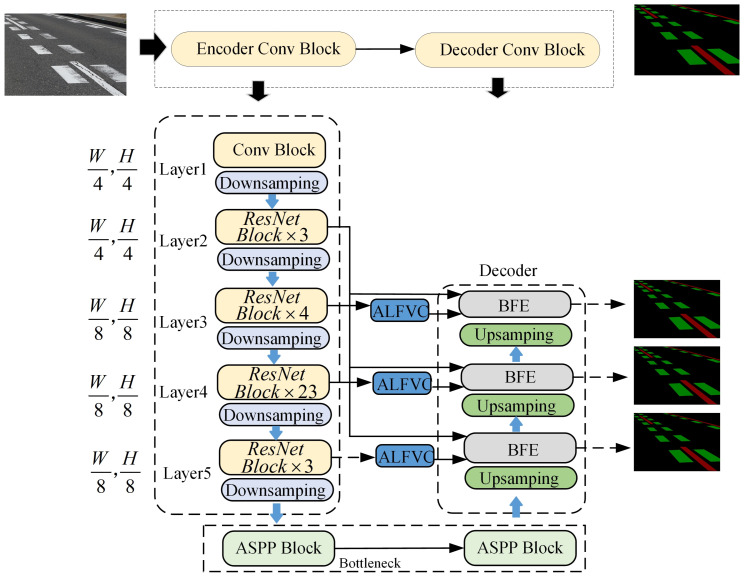
Detailed network structure diagram of the proposed network for region segmentation. “ALFVC” is the proposed network structure for asymmetric large field-of-view feature extraction. “BFE” is the proposed network structure for edge feature information enhancement.

Current detection methods are limited to identifying the presence of damage to road signs and cannot accurately quantify the extent of the damage. To address this gap, this paper proposes a novel method for quantifying the degree of road markings damage. Figure 1 illustrates the overall framework for the proposed degree of road markings damage detection method. This method consists primarily of two components. The first stage involves segmenting the residual road marking pixel points after damage. The second component is used to segment the original region of the damaged road markings, which is required to obtain the complete region of the road markings when it is not damaged during the segmentation process, and this part is crucial for the detection of the degree of damage of the marking. Therefore, an asymmetric large field-of-view contextual feature integration module is proposed to extract richer contextual information. Additionally, a boundary information enhancement module is introduced to learn boundary features of marking, facilitating better segmentation of the original regions when damage occurs. By segmenting these two parts, the area of the pavement marking before and after the breakage can be obtained (in the image, the area is obtained by counting the number of pixel points). Then, the difference between the areas before and after damage is calculated, and the ratio relative to the original area is used to determine the degree of damage to the pavement marking. Figure 2 presents a detailed network architecture diagram for the region segmentation method introduced in this study.

Figure 2 shows the structure of the network designed for region segmentation. Unlike other segmentation networks, the network structure designed in this paper uses multi-scale feature extraction and fuses feature information from different feature layers, which is more conducive to the model to be able to capture more details of the image, which helps segment the road markings and background in the image more accurately.

### 3.1. ALFVC Design

**Figure 3:**
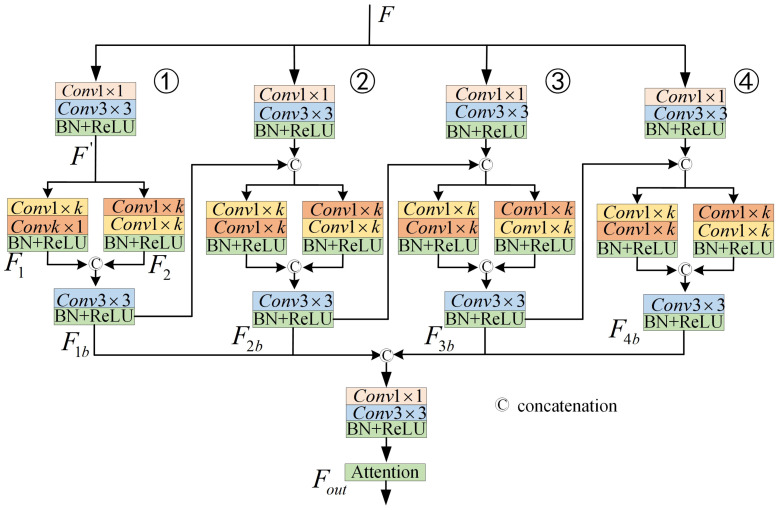
Asymmetric feature extraction network structure.

Contextual feature information is pivotal for the segmentation of road marking areas, as it provides the model with global features and a broad field of view, enabling the model to consider the overall road layout and structure, thereby improving the accuracy and consistency of the segmentation. Particularly, in cases of missing or blurred road marking sections, global contextual features can guide the extension and connection of markings, reducing the likelihood of misjudgment and erroneous segment. Moreover, the adaptability of large-view features to environmental variations, such as changes in illumination, occlusions, and diverse weather conditions, significantly enhances the robustness and reliability of the segmentation process. The asymmetric large field-of-view contextual feature extraction module proposed in this paper effectively extracts and integrates contextual marking feature information across multiple scales, facilitating contextual reasoning and precise localization of road markings. In this study, multiple asymmetric convolutions are employed to achieve a larger field of view for contextual feature extraction, enhancing the network’s ability to capture relevant information from diverse perspectives. Asymmetric convolution captures features through directional convolution operations, enabling more efficient extraction of features across various orientations and significantly enhancing the feature extraction capabilities of convolutional neural networks. Furthermore, road markings typically exhibit slender and continuous shapes. Asymmetric convolution can separately extract features in horizontal and vertical directions, allowing for a more accurate capture of the morphological characteristics of these markings. Figure 3 depicts the structure of the proposed asymmetric contextual feature information extraction network.

The structure of the proposed asymmetric large-view contextual feature extraction network consists of four parallel branches, each of which corresponds to a different size of sensory field feature information. The structure of branch ① in Figure 3 is elaborated in detail. The specific expressions are as follows:

(1) $$F^{\prime }=\partial (Conv_{3\times 3}(Conv_{1\times 1}(F)))$$

A convolutional kernel of 3×3 and 1×1 was used for feature extraction at the very beginning of each branch, which was carried out to make each branch produce more distinguishable feature information. In Equation (1), Conv denotes the convolution operation, and its subscript corresponds to the size of the convolution kernel. F denotes the input feature information. ∂ denotes the batch normalization and ReLU operation. $F^{\prime }$ denotes the feature information after convolution.

Given the slender and continuous nature of road markings, vertical directional feature information is of greater significance. Consequently, two additional asymmetric convolutional blocks are incorporated in parallel within each branch path: one dedicated to extracting vertical directional features and the other to horizontal directional features. The final features extracted from these two pathways differ structurally; however, they may offer complementary insights as they can be further refined through convolutional operations in orthogonal directions. The specific formulation is detailed as follows:

(2) $$F_{1}=\partial (Conv_{K\times 1}(Conv_{1\times K}(F^{\prime })))$$

(3) $$F_{2}=\partial (Conv_{1\times K}(Conv_{K\times 1}(F^{\prime })))$$

where $F_{1}$ and $F_{2}$ are denoted as the feature information extracted using asymmetric convolution in vertical and horizontal directions, respectively. After obtaining the feature information in different directions, a convolution of 3×3 and 1×1 is utilized to further fuse the feature information in both dire.

(4) $$F_{1b}=\partial (Conv_{3\times 3}(Concat(F_{1},F_{2})))$$

where Concat(,) denotes for channel splicing. $F_{1b}$ denotes the feature information of the first branch fusion.

Given that marking appear smaller as their distance from the camera increases and larger as they approach the camera, a single fixed field of view fails to account for marking at varying distances from the camera. Consequently, we employed four asymmetric convolutional blocks with varying receptive fields in parallel to capture more comprehensive information on both upper and lower features (that is, each branch convolution kernel size is 3, 5, 7, 9). Through the fusion of asymmetric structures across these four branches to extract feature information, we obtain richer contextual features. However, this process may also introduce unwanted noise. To address this issue, we employed an attention module [40] to refine and output the fused feature information.

(5) $$F_{fusion}=\partial (Conv_{3\times 3}(Conv_{1\times 1}(Concat(F_{1b},F_{2b},F_{3b},F_{4b}))))$$

(6) $$F_{out}=attention(F_{fusion})$$

The $F_{fusion}$ in Equation (5) denotes the features after fusion of the 4 branches. The $F_{out}$ in Equation (6) denotes the final output of the module.

### 3.2. BFE Design

**Figure 4:**
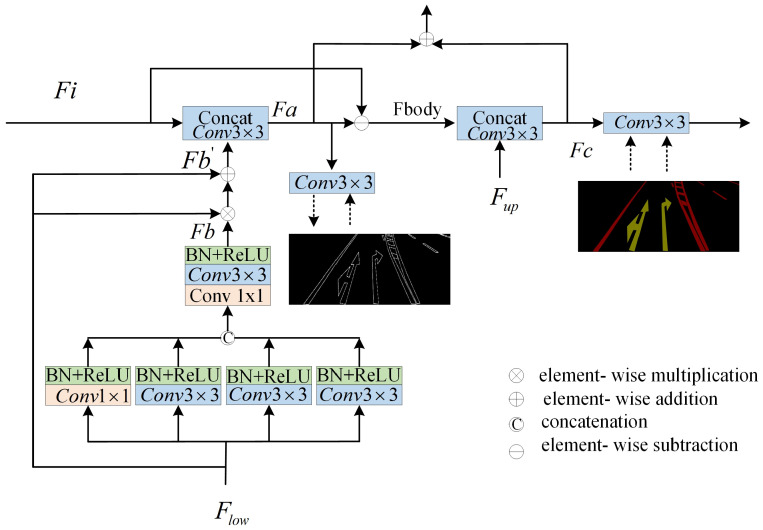
Boundary feature enhancement network structure.

This study emphasizes the precise segmentation of the original marking area from damaged road markings, with a particular focus on segment the complete marking region as it existed prior to damage. This is crucial for assessing the extent of damage to the markings, with boundary features being instrumental in accurately delineating the boundaries of the markings within the image. In the process of segment damaged images, the accurate identification and segmentation of these boundaries are pivotal for segment the original state of the markings. Boundary features are essential for conveying information about the structure and shape within the image. Learning these features aids the model in comprehending the shape of the road markings. Furthermore, retaining boundary features is crucial to preserving the details within the image, thereby maintaining its realism and the level of detail. In this paper, we introduce a supervised BFE module designed to enable the model to learn both the boundary and shape features of the markings throughout the training process. Figure 4 depicts the detailed network architecture of the proposed BFE module.

The proposed BFE module leverages the boundary feature information of road markings. In neural network architectures, boundary feature information is considered a low-level feature and is typically found within the first two layers of the network. In this study, the BFE structure utilizes the second convolutional layer of the backbone network to extract the boundary feature information of road markings ($F_{low}$ in Figure 4 is from “Layer2” in Figure 2). Since the second layer of the convolutional neural network is primarily focused on extracting low-level features such as textures, edges, and corner points from the input image, these features are influenced by various patterns, colors, and brightness variations. Consequently, there may be interference and cross-influence among numerous low-level features in the second layer, leading to a substantial amount of noise in the output feature map. To address this issue, we employed four convolutional kernels of varying scales with different receptive fields (1, 3, 5, and 9) to further extract the edge feature information. These convolutional kernels are deployed through a parallel branching structure, with each branch comprising a convolutional layer, a batch normalization (BN) layer, and a ReLU nonlinear activation to efficiently capture and integrate multi-scale edge features. This process is depicted in Equations (7)–(11).

(7) $$g_{1}=\partial (Conv_{1\times 1}(F_{low}))$$

(8) $$g_{2}=\partial (Conv_{3\times 3}(F_{low}))$$

(9) $$g_{3}=\partial (Conv_{3\times 3}(F_{low}))$$

(10) $$g_{4}=\partial (Conv_{3\times 3}(F_{low}))$$

(11) $$Fb=\partial (Conv_{3\times 3}(Conv_{1\times 1}(Concat(g_{1},g_{2},g_{3},g_{4}))))$$

Equation (11) is used to fuse features from the four branches. We denote the enhanced shallow edge features as Fb.

(12) $$Fb^{\prime }=(F_{low}⊗Fb)⊕F_{low}$$

In Equation (12), ⊗ represents the multiplication of elements. ⊕ denotes the addition of elements. $F_{low}$ denotes the shallow input features. $Fb^{\prime }$ denotes the fused features. After obtaining the enhanced shallow feature information, we further used the convolution operator to fuse the enhanced shallow information to obtain the refined edge features, which is shown by Equation (13).

(13) $$Fa=\partial (Conv_{3\times 3}(Concat(Fb^{\prime },Fi)))$$

Fa is denoted as refined edge features, and the refined learning is performed by supervising the Fa using the edge graph in the label. In addition, after the learning of the edge features, the information of the labeled body features in addition to the edge features is considered Fbody, which is obtained by the process utilizing Fi minus Fa, which is shown by Equation (14).

(14) Fbody=Fi−Fa

In addition, the fused features from the previous step may be noisy and further will be fused with more advanced feature information to obtain more refined edge features, and this process we likewise utilize really labels for supervised learning.

(15) $$Fc=\partial (Conv_{3\times 3}(Concat(Fbody,F_{up})))$$

The representation of $F_{up}$ in Equation (15) denotes the feature map after upsampling, as shown in Figure 2.

### 3.3. Loss Function

In our designed detection network has three branches for predicting segmentation, and in each branch there are three different predictions, each of which corresponds to a different component of the labeled line, i.e., $F_{Fedge}$, $F_{Fbody}$, and $F_{pre}$, where $F_{Fedge}$ and $F_{Fbody}$ predict the edge feature information of the labeled line, and $F_{pre}$ predicts the final segmentation result. We constructed a joint loss function for each branch for supervising their learning.

(16) $$\begin{array}{c} loss=\gamma_{1}L_{Fedge}(F_{Fedge},Ge)+\gamma_{2}L_{Fbody}(F_{Fbody},Gb)\\ +\gamma_{3}L_{pre}(F_{pre},Gu) \end{array}$$

where Gu denotes the original ground truth, Gb denotes Gu in the premise of removing the boundary of the label so that the boundary will not affect the calculation of the loss function and model learning during the training process, and Ge denotes only retained the boundary Gu mask value. We use $\gamma_{1}$, $\gamma_{2}$, $\gamma_{3}$ to denote the importance of different prediction results. In our experiments, $\gamma_{1}=\gamma_{2}=\gamma_{3}=1$. The final loss function of the model is the sum of the loss functions of each branch. In our experiments $L_{Fbody}(F_{Fbody},Gb)$ and $L_{pre}(F_{pre},Gu)$ use cross-entropy loss, and $L_{Fedge}(F_{Fedge},Ge)$ uses the Dice Loss function [41].

## 4. Experimental Verification and Analysis

This section begins by introducing relevant metrics used for model evaluation. Subsequently, we provide a detailed description of the two datasets introduced in this study: the road marking Pixel Point Segmentation dataset and the road marking Region Segmentation dataset. Additionally, we conducted experimental comparisons between the proposed improved network and other networks using our datasets to validate the performance of our method. Finally, we assessed the detection performance of our proposed detection method on the dataset, evaluating road markings with varying degrees of damage.

### 4.1. Model Evaluation Indicators and Experimental Environment

In order to accurately evaluate the detection performance of the proposed method in road markings, the intersection and merger ratio (*IoU*) for each category, the total pixel segmentation mean intersection and merger ratio (*mIoU*), and the predicted total pixel accuracy and $\text{Macro-}F_{1}\text{-score}$, the specific formula for the metrics is as follows:

(17) $$PA=\frac{TRP}{TNP}$$

(18) $$IoU_{i}=\frac{TP_{i}}{TP_{i}+FP_{i}+FN_{i}}$$

(19) $$mIoU=\frac{1}{N}\sum_{i=1}^{N}IoU_{i}$$

where *TRP* denotes the correctly predicted pixel point and *TNP* denotes the total number of pixel points in the label. In Equations (17) and (18), $TP_{i}$ represents the true positive of class i, $FP_{i}$ represents the false positive of class i, and $FN_{i}$ represents the false negative of class i. N is the number of classes.

(20) $$F_{1}=\frac{1}{N}\sum_{i=1}^{N}F_{1}{\text{-score}}_{i}$$

$F_{1}$ is used as an evaluation metric for multi-category classification tasks. It measures the average performance of the classifier on different classes, ignoring the imbalance of classes, so that in the case of class imbalance, it better reflects the average performance of the model on each class without being dominated by the performance of the majority class. In addition, for the evaluation of the model we have selected Mean absolute error (*mAE*) and balance error rate (*mBER*).

At the same time, the damage degree of the road markings is calculated after the number of pixels of the marking line is obtained by using the pixel point segmentation formula and the area segmentation respectively. In this paper, the calculation method of marking damage rate is given as shown in Equation (21).

(21) $$Rate=\frac{p2-p1}{p2}\times 100\%$$

where *p2* is denoted as the total number of pixel points of the labeled line obtained using area segmentation, and *p1* is denoted as the total number of pixel points obtained using the pixel point segmentation method.

All experimental models in this paper were carried out on a Windows 11 computer with an Intel(R)Core(TM), i9-14900K, 3.20 GHz processor and a 24 G sized 4090 graphics card. All code was carried out in PyTorch framework using python3.8.

### 4.2. Datasets

**Figure 5:**
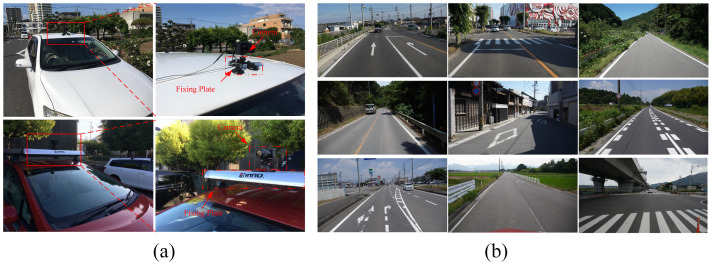
Data collection. (**a**) The equipment used for data collection and the installation position of the devices. (**b**) Data images collected under different scenarios and various environmental conditions.

In this study, we constructed a comprehensive large-scale road marking dataset tailored for deep learning model training and evaluation. The dataset comprises two distinct components: labels for the residual pixel point segmentation of damaged road markings and labels for the complete segmentation of the marking area. For the damaged road marking residual pixel point segmentation, the dataset includes over 2500 training images, each meticulously annotated by hand. Similarly, for region segmentation, we generated 3200 training images, each meticulously annotated to produce 3200 corresponding training labels. In this study, the training data images were collected from real-world environments at various time points, ensuring a diverse and representative dataset. The data collection methodology is illustrated in Figure 5a, utilizing a Sony α6500 model camera to capture high-quality images. Notably, the original label images were procured by the local government of Mie Prefecture, Japan, ensuring the authenticity and accuracy of the dataset. All the images in the dataset were captured on roads using the method shown in Figure 5a. Therefore, all images are forward-facing, as illustrated in Figure 5b. To ensure image clarity, all photographs were taken under sunny weather conditions. The dataset exhibits diversity, encompassing various road conditions, types of markings (with a focus on arrow indicators, zebra crossings, lane edge lines, guide lines, and speed bump lines), and levels of damage, ensuring broad adaptability for model training and evaluation.

#### 4.2.1. Pixel Point Segmentation Data

**Figure 6:**
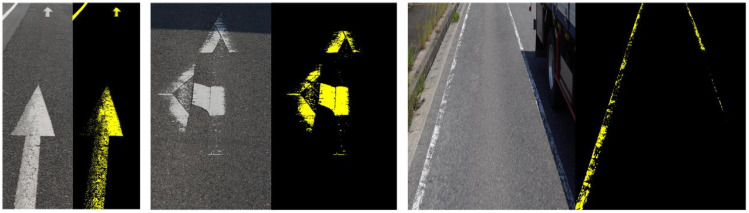
Pixel point segmentation data annotation.

To facilitate the segmentation of residual markings, a specialized dataset was developed for the segmentation of residual road marking pixel points. A binary image format was employed to represent the residual road markings on the ground, serving as the ground truth for the segmentation process. Figure 6 illustrates an example of the annotation process for residual markings. In our provided annotations, a pixel point is labeled as 1 if it is covered by the paint of the marking line, and 0 otherwise. Specifically, if a location was initially covered by the marking line paint but became uncovered after damage, the corresponding pixel is set to 0. Our dataset annotations do not include a detailed delineation of the road markings line, focusing instead on broader segmentation categories. The annotations in this dataset categorize pixels into two classes: background and road markings, without further subdivision into detailed road markings.

#### 4.2.2. Regionally Segmented Datasets

**Figure 7:**
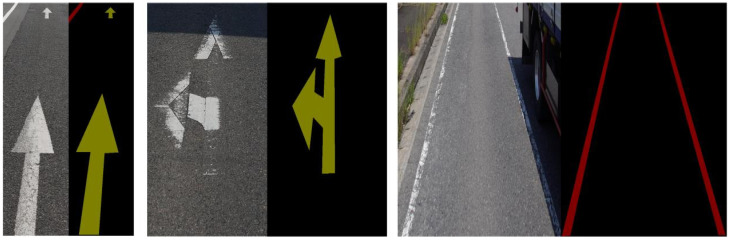
Annotation of regionally segmented data.

To delineate the regions of the original marking, we constructed a dataset for road marking region segmentation, mirroring previous methodologies. As illustrated in Figure 7, annotations of the road marking regions are stored using binary images, which clearly delineate the road markings areas. Our dataset includes annotations of the marking regions, representing the areas as they were prior to any damage. In essence, we annotate the original conditions of the markings. Post-training, the proposed network demonstrates the capability to reconstruct the original region of the marking, even in instances of physical degradation. Notably, within the region segmentation dataset, road markings are categorized into four distinct classes, each annotated with a unique color in the binary images.

### 4.3. Experimental Comparison of ALFVC and BFE Component Performance

To further investigate the individual contributions of the two proposed components for road marking segmentation, we conducted ablation experiments to assess the specific role of each within the network. Notably, the base model employed in our experiments is the Deeplabv3 architecture, utilizing ResNet101 as the backbone. ResNet101 utilizes residual learning to address the vanishing gradient problem, enabling the effective training of very deep networks. With 101 layers, it balances model depth and computational efficiency, making it well-suited for feature extraction tasks in image segmentation and other computer vision applications. We specifically utilized only the down sampling component of this backbone network. Furthermore, all pre-trained models of the backbone network employed identical pre-training weights to ensure consistency. Table 1 presents the impact on segmentation performance when various components are integrated into the base architecture.

**Figure 8:**
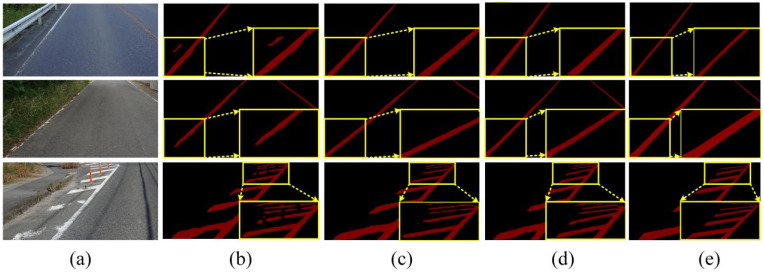
Visualization of road marking segmentation performance of different components in different scenarios. The yellow boxes indicated by arrows in the image are local zooms. (**a**) Input image. (**b**) Baseline. (**c**) Baseline + BFE. (**d**) Baseline + BFE + ALFVC. (**e**) Ground truth.

Initially, we evaluated the performance metrics of the base model on our provided dataset. Subsequently, we integrated the proposed BFE module into the base model. As indicated in Table 1, the addition of the BFE module enhances the mIoU by 5.515 percentage points: mBER by 0.0056 and mAE by 0.22. Additionally, various degrees of improvement were observed in the PA performance. These findings demonstrate the superior performance of the proposed BFE module in enhancing edge feature information for segmenting damaged labeled lines. Finally, the integration of the proposed ALFVC module with the BFE module resulted in a further increase in mIoU by 0.346 percentage points, along with improvements in other performance metrics. Although the proposed ALFVC module does not yield as substantial numerical performance gains as the BFE module, it offers significant visual improvements, as evidenced by the segmentation results in the third row of Figure 8 (from top to bottom). In the top part of the image, a red obstacle in the original image obscures the labeled line, causing discontinuities in the segmentation. However, the segmentation results using the ALFVC module accurately segment this area. Table 2 illustrates the IoU value comparisons between the proposed components and the base model across different segmentation categories. The table clearly indicates that both proposed components achieve superior performance in each category.

**Figure 9:**
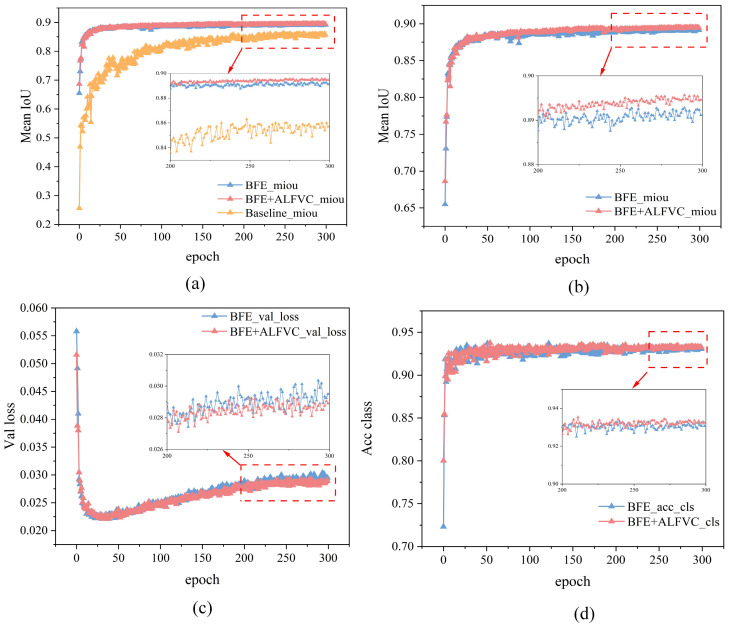
The road marking segmentation performance of various methods across different scenarios. (**a**) The mIoU comparison between the baseline model and the models with BFE and BFE + ALFVC modules, respectively. (**b**) The mIoU comparison of the models with BFE and BFE + ALFVC modules, respectively. (**c**) The validation loss during the training process for the models with BFE and BFE + ALFVC modules, respectively. (**d**) The category accuracy during training for the models with BFE and BFE + ALFVC modules, respectively. The closer the “Mean IoU” and “Acc class” values are to 1, the better the model’s performance. The closer the “Val loss” value is to 0, the better the model’s performance.

Figure 9 presents a comparison of various metrics between the proposed method and the base model during the training process. It is evident that both the Baseline + BFE and Baseline + BFE + ALFVC configurations significantly outperform the base model in terms of mIoU metrics throughout the training. The figure illustrates the comparison of performance indices for Baseline + BFE and Baseline + BFE + ALFVC during training. It is clear that the model incorporating both BFE and ALFVC exhibits superior detection performance across all metrics. The primary reason for this improvement is that the BFE module enhances the model’s ability to learn edge shape features of the marking during training, allowing for the accurate segmentation of the original areas of damaged markings. Additionally, the ALFVC module allows the model to capture more contextual features from a larger receptive field, thereby further enhancing performance. The four parallel asymmetric convolutional modules are particularly effective in detecting bar-shaped markings.

### 4.4. Experimental Comparison with Other Segmentation Models

To validate the efficacy of the proposed method, we conducted a comprehensive experimental comparison across various methodologies. In our experimental setup, we selected 11 distinct segmentation networks for comparative analysis, with selection criteria including the following: (1) classical image segmentation models and (2) state-of-the-art performance in specific domains. Specifically, the networks selected include FCN32s, FCN16s, FCN8s, FCN, CGNet [42], DeepLabv3, LEDNet [43], PSPNetunet [32], ViT [44], and swim [44]. Additionally, we adapted the feature extraction backbone of these networks to assess their performance in road markings segmentation under varying backbone architectures. All models were trained on a dataset curated by us. Notably, the method proposed herein is based on the ResNet101 architecture with specific modifications. Table 3 illustrates the performance metrics of various methods in road markings region segmentation. As evidenced by the data in Table 1, segmentation models utilizing the VGG architecture exhibit significantly lower performance compared to those employing ResNet50 and ResNet101 frameworks. Furthermore, the network region segmentation method proposed in this study outperforms all other models across all segmentation metrics.

Table 3 presents a comparison of segmentation results between proposed method and 11 other methods on damaged road markings. It is evident that our proposed method outperforms all others across the four detection metrics presented. Additionally, we conducted a comparison of the mIoU, PA, mBER, and mAE performance for each method across different categories of road markings. Our method excels in all evaluated aspects, achieving the highest PA of 99.34, the highest mIoU at 89.44, the lowest mBER of 0.03, and the lowest mAE of 0.98. These results highlight our method’s superior pixel-level accuracy, excellent segmentation overlap, precise boundary predictions, and minimal error rate.

Table 4 details the IoU performance comparison among various methods across distinct categories. The experimental results indicate that our proposed method demonstrates optimal detection performance across all categories in terms of IoU. Especially, the performance is especially outstanding in category 3 and category 4 damaged markings, reaching 93.87 in category 3 damaged markings and 86.45 in category 4 damaged markings, which is much higher than other segmentation methods. This verifies its excellent segmentation ability in dealing with complex scenes of real roads.

**Figure 10:**
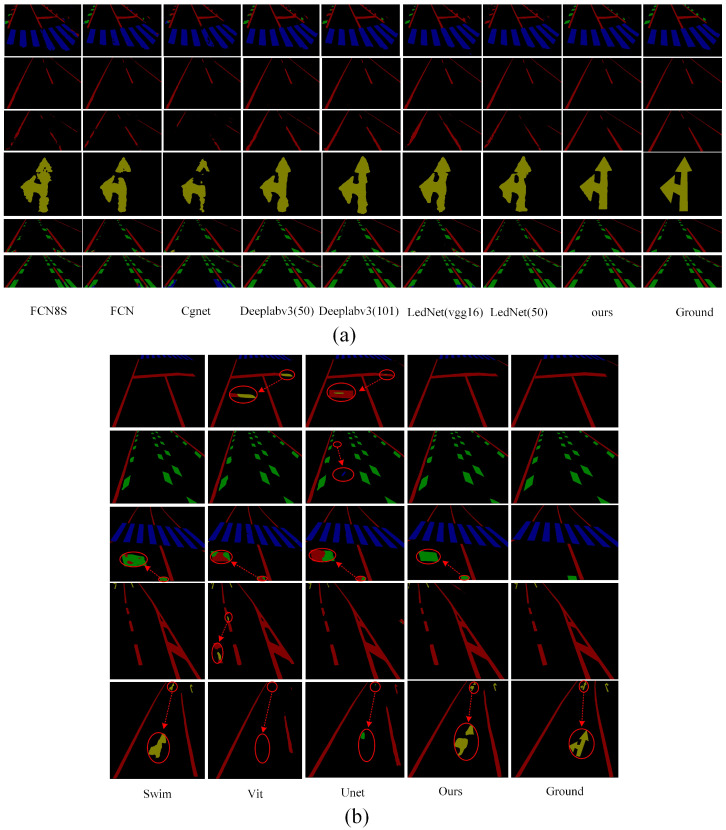
(**a**) Classic CNN segmentation method. (**b**) Based on Transformer and our method.

To provide a more intuitive demonstration of the actual performance of various methods for road marking segmentation, we selected images from diverse scenes and environments for comparative analysis. We selected the top eight models with the highest mIoU values for segmenting damaged markings across different scenes. Figure 10 illustrates the visualized results of segmentation using various methods.

Figure 10a clearly demonstrates that our proposed method continues to accurately segment the original regions of road markings, even in cases of severe damage. This further substantiates the effectiveness of our method in region segmentation, particularly in accurately delineating the original regions of damaged markings. The primary factor contributing to this performance is the ALFVC module, which effectively extracts rich contextual feature information. The four distinct feature scales provided by the ALFVC module facilitate the accurate localization of road markings, including those not easily passed over. Additionally, the proposed BFE module enhances the model’s ability to accurately capture edge features, thereby enabling precise segmentation of the original marking areas, even in the presence of damaged markings. The image in the fifth row of Figure 10 (counting from top to bottom) clearly illustrates the capability of our proposed method to precisely segment the original regions from the damaged markings. As illustrated in Figure 10b, our method consistently outperforms existing approaches (Swin, ViT, and UNet) in accurately detecting and restoring damaged road markings. Unlike prior methods which often fail to capture fine-grained discontinuities or misclassify ambiguous regions (highlighted by red circles), our approach yields more precise segmentation boundaries and superior structural completeness. Particularly, in complex cases involving occlusion or blurred patterns, our model exhibits better robustness and semantic consistency, closely matching the ground truth.

### 4.5. Test Experiments on the CeyMo Dataset

To verify the generalization capability of the proposed method, we evaluated it on the open-source CeyMo dataset [45]. The CeyMo dataset encompasses a substantial number of road marking images, including various types and shapes such as solid, dashed, double-solid, and crosswalk markings. These images are ideal for the training and evaluation of road markings detection, recognition, and classification, providing a crucial database for research into traffic safety and autonomous driving systems. In all models, the input image size was set to 512 × 512 pixels and the initial learning rate of the model was set to 0.002 with decay per epoch. In addition, to ensure a fair evaluation of the model, we used two macro F1 scoring metrics from the CeyMo paper.

**Figure 11:**
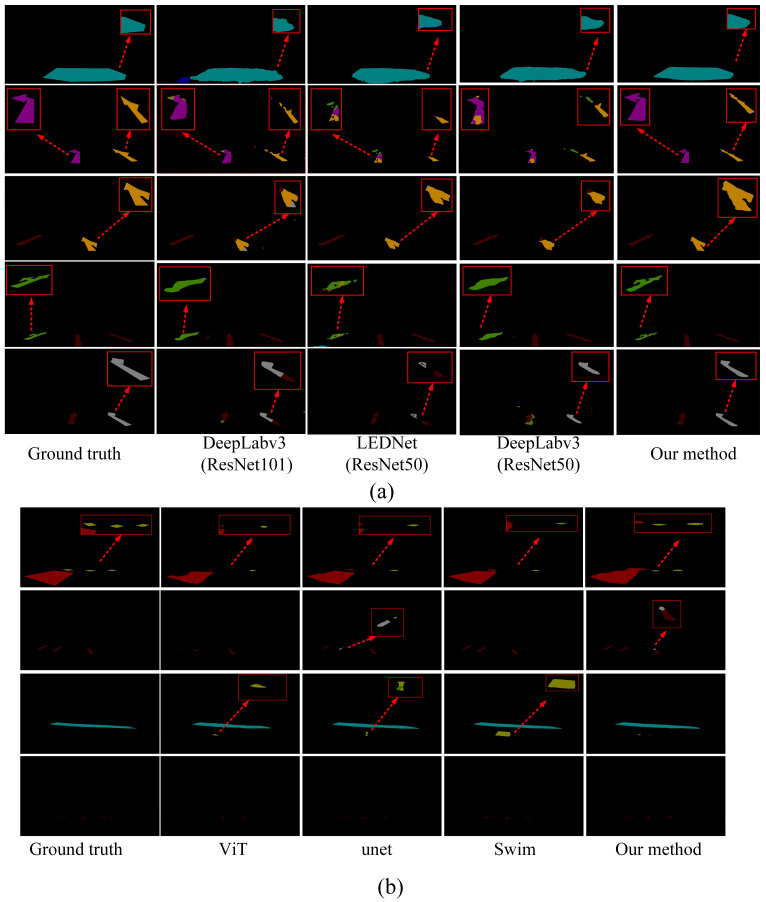
(**a**) The traditional CNN-based segmentation method. (**b**) The Transformer and Unet-based segmentation method.

Table 5 compares the performance of our proposed method with several state-of-the-art segmentation models on the open-source CeyMo dataset. The results of Mask-RCNN are taken from the original paper [44]. Although our method achieves a slightly lower F1 score (97.13) compared to the best-performing Swin segmentation model (97.38), it significantly outperforms all other competitors, including the Transformer-based ViT model. Figure 11 presents the visual prediction results under varying lighting conditions, highlighting the robustness of different methods.

As illustrated in Figure 11, the proposed method exhibits substantial improvements over conventional segmentation models. While it slightly lags behind the Swin model in terms of segmentation accuracy, it clearly surpasses the ViT model, highlighting its effectiveness among Transformer-based approaches.

### 4.6. Calculation of the Degree of Damage

**Figure 12:**
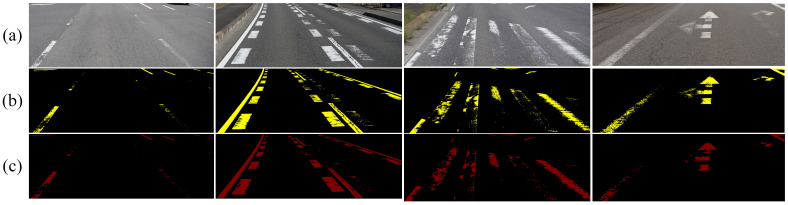
Visualization of case histories for pixel point segmentation, where (**a**) denotes the input image, (**b**) denotes the label corresponding to the input image, and (**c**) denotes the model predicted segmentation.

In the proposed method for road marking damage detection, the residual marking area post-breakage was obtained using a pixel segmentation technique. Specifically, we utilized the DeepLabv3 network to directly segment the damaged markings. Table 6 presents a series of performance index parameters of the DeepLabv3 network during the segmentation validation process. In the pixel segmentation task, no modifications were made to the network model. The original network model was used to segment the pixel points of the damaged road markings. As can be clearly seen in Table 6, although we used the original network, the pixel segmentation mIoU still reaches 94.430. Some visualized case studies of pixel point segmentation are shown in Figure 12.

**Figure 13:**
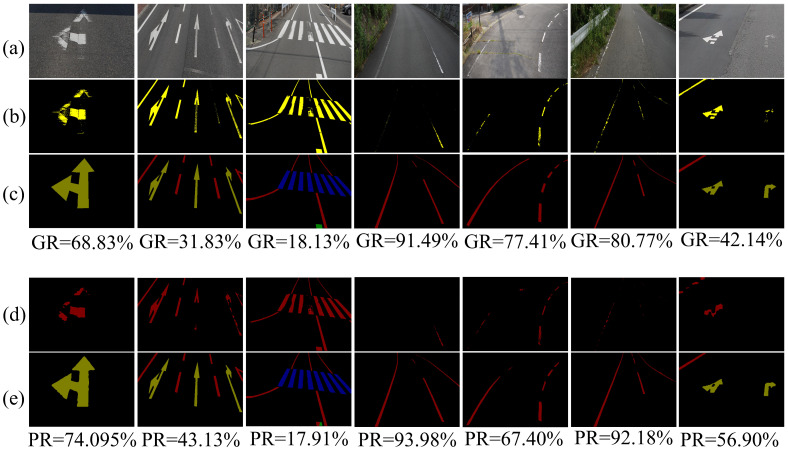
Acquisition of the degree of damage. (**a**) Input image. (**b**) Input image with corresponding pixel segmentation labels. (**c**) Input image with corresponding region cut labels. (**d**) Predicted pixel segmentation. (**e**) Predicted region cut. Here, GR represents the marking line breakage rate calculated using the two labeling files, and PR represents the marking line breakage rate predicted using the two models. It should be noted that both GR and PR are calculated using Equation (21).

In our proposed method, the degree of damage is determined by comparing two segmented regions to obtain the total number of pixel points, thereby assessing the extent of road marking breakage. Figure 13 demonstrates the effectiveness of our proposed method in assessing the degree of damage. To compare the detection accuracy more intuitively, Figure 13 also provides the actual degree of damage in the image, which is obtained by using pixel segmentation to separate labels corresponding to the image with the labels of the region segmentation.

The minimum error in damage rate detection is 0.22% (GR = 18.13%, PR = 17.91%), while the maximum error is 14.76% (GR = 42.14%, PR = 56.90%). From Figure 13c,e (i.e., the real region-segmented labels and the region-predicted segmented images), it is evident that the region-predicted segmented images are the closest to the real label values and exhibit effective segmentation for damaged regions. However, from Figure 13b,d (i.e., predicted pixel points and actual standard pixel points), it is apparent that there are discrepancies between the predicted pixel points and the actual standard pixel points. The reason for this discrepancy lies in the complexity and difficulty of the labeling process for the pixel point segmentation model, which necessitates labeling every white pixel point, a task that is challenging to execute uniformly. Since the same RGB pixel value may be classified into different categories (background or marking) in various images and under different light intensities, a considerable amount of noise exists in the label file. We contend that this noise is the primary cause of the significant discrepancy between the final predicted degree of damage and the degree of damage recorded in the labeling file. Overall, the method proposed in this paper is effective in determining the degree of damage of road markings.

Further, to verify the efficiency of the proposed method in real detection tasks, we evaluated the inference speed of the model on a single NVIDIA RTX 4090 GPU. In the experiments, the input image resolution is 512 × 512, and the test results are shown in Table 7, which demonstrate the performance metrics of the model’s average inference time and frame rate (FPS) under this hardware configuration.

## 5. Limitations and Future Research Directions

In this paper, the degree of damage of the markings was calculated using all the pixel points of the marking in the entire image. However, for certain localized cases of severe breakage in the image, the pixel points in these regions account for a small proportion of the pixel points of the whole marking line, possibly resulting in the degree of damage calculated using the proposed method being low. Therefore, the proposed method has some limitations in dealing with marking lines with severe localized damage. In addition, a large number of labels with noise in the pixel point segmentation stage affects the model’s segmentation capability, which, in turn, affects the degree of damage to the final computed marking. Therefore, future research will address the above three issues in depth and improve them. Therefore, in our future work, we will solve the three problems by converting the front view to a top view and combining the target detection algorithm to detect the localized marking.

## 6. Conclusions

In this paper, the focus was on detecting the degree of damage of road markings from road images in order to alert road maintenance personnel to repair severely damaged marking areas in a timely manner. Aiming at the characteristics of elongated road markings and the shortcomings of existing methods that cannot obtain global contextual feature information, an asymmetric large-view contextual feature module is proposed. This module can not only acquire contextual feature information, but also extract multi-scale marking feature information. In addition, an enhanced edge module was designed to enhance the learning of the edges of the marking line features so that it is easier to segment the complete region from the damaged marking line. The experimental results of different methods were compared on our constructed dataset and the open-source CeyMo dataset, and the proposed method performs optimally in terms of detection performance. Validated with real labeling data, the proposed method accurately detects the degree of damaged markings in an image. In order to meet the requirements of real-time detection, in the future, we will consider using a lighter segmentation model or introducing a more efficient decoding structure, using model pruning or knowledge distillation and other technologies to significantly improve the running speed while maintaining accuracy, so as to better meet the needs of real-time application scenarios.

## Figures and Tables

**Table 1:** Comparison of segmentation effect of different components.

Baseline	+BFE	+ALFVC	mIoU	mBER	mAE	PA
ResNet101	-	-	83.58	0.03	1.21	99.13
	√	-	89.09	0.02	0.99	99.33
	-	√	88.17	0.03	0.99	99.33
	√	√	89.44	0.02	0.98	99.33

**Table 2:** Comparison of the segmentation effects of different components.

Method	Backbone	Background	Lines	Zebra Crossings	Guide Lines	Speed Bump Lines
Baseline	ResNet101	99.12	79.20	78.79	77.55	83.22
+BFE	ResNet101	99.33	84.05	82.17	94.06	85.85
+ALFVC	ResNet101	99.33	84.03	83.21	93.24	86.13
+BFE + ALFVC	ResNet101	99.33	84.11	83.43	93.87	86.45

**Table 3:** Comparison of different advanced methods.

Method	Backbone	PA	mIoU	mBER	mAE
FCN32s	VGG16	98.08	60.31	0.10	4.81
FCN16s	VGG16	98.50	66.37	0.07	3.35
FCN8s	VGG16	98.75	70.56	0.06	2.70
FCN	ResNet50	98.80	73.86	0.05	2.38
CGNet	VGG16	98.57	64.37	0.67	3.42
DeepLabv3	ResNet50	99.04	80.57	0.03	1.33
DeepLabv3	ResNet101	99.13	83.58	0.03	1.21
LEDNet	VGG16	98.89	76.64	0.05	2.47
LEDNet	ResNet50	98.93	76.43	0.06	2.50
PSPNet	ResNet50	98.55	73.71	0.07	3.22
PSPNet	ResNet101	98.59	71.93	0.07	3.12
UNet	---	99.17	85.61	0.03	1.24
Swin	Transformer	99.27	88.18	0.03	1.03
ViT	Transformer	99.10	83.11	0.04	1.61
ours	ResNet101	99.34	89.44	0.03	0.98

**Table 4:** Comparison of metrics of different advanced methods on IoU.

Method	Backbone	Background	Lines	Zebra Crossings	Guide Lines	Speed Bump Lines
FCN32s	VGG16	98.19	61.88	44.89	48.42	48.15
FCN16s	VGG16	98.64	69.17	52.85	58.45	52.75
FCN8s	VGG16	98.90	73.66	57.87	64.36	58.02
FCN	ResNet50	98.94	74.56	62.60	72.66	60.54
CGNet	VGG16	98.77	70.11	53.74	48.56	50.64
DeepLabv3	ResNet50	99.04	77.60	74.88	73.16	78.20
DeepLabv3	ResNet101	99.12	79.20	78.79	77.55	83.22
LEDNet	VGG16	98.92	74.75	69.31	63.76	76.45
LEDNet	ResNet50	99.00	76.04	70.43	62.32	74.32
PSPNet	ResNet50	98.73	68.46	62.90	61.33	77.11
PSPNet	ResNet101	98.76	70.02	59.51	62.06	69.28
UNet	---	99.18	79.50	82.65	81.47	85.27
Swin	Transformer	99.27	81.62	87.98	88.15	83.87
ViT	Transformer	99.10	78.50	81.50	79.51	76.91
Ours	ResNet101	99.33	84.11	83.43	93.87	86.45

**Table 5:** Comparison of metrics of different advanced methods on CeyMo. The models are denoted as follows: M1: DeepLabv3 (ResNet-101), M2: DeepLabv3 (ResNet-50), M3: LEDNet (ResNet-50), M4: Mask R-CNN (ResNet-50), M5: ViT, M6: U-Net, and M7: Swin Transformer.

Class	M1	M2	M3	M4	M5	M6	M7	Ours
**SA**	84.37	79.77	95.16	88.33	95.23	92.26	95.95	95.60
**LA**	82.96	85.83	73.40	74.36	94.75	89.61	95.53	95.59
**RA**	86.07	90.33	89.22	90.40	96.77	94.67	97.61	96.83
**SLA**	85.47	86.83	85.14	89.47	96.41	94.52	96.27	95.88
**SRA**	72.71	85.09	68.31	66.67	92.52	83.63	92.65	92.15
**DM**	90.07	90.66	90.43	91.05	97.20	95.09	97.66	96.83
**PC**	99.00	99.01	97.92	96.86	99.21	98.24	99.53	99.72
**JB**	95.47	97.66	95.06	96.83	99.17	97.97	99.50	99.59
**SL**	93.46	96.94	92.47	94.34	99.08	97.21	99.48	98.86
**BL**	91.53	93.64	91.34	91.26	97.47	95.29	97.97	97.48
**CL**	92.78	95.92	91.63	92.31	99.06	96.28	99.06	97.67
**F1**	88.617	91.06	88.20	88.33	96.99	94.07	97.38	97.13

**Table 6:** Pixel segmentation performance metrics.

Method	Backbone	Number of Pictures	Background (IoU)	Marking (IoU)	mIoU
DeepLabv3	Resnet101	253	99.60	88.76	94.43
UNet	---	253	99.36	82.87	91.11
Swin	Transformer	253	99.38	83.39	91.38
vit	Transformer	253	99.32	82.27	90.80

**Table 7:** Detection efficiency test (‘1’ denotes the model used to segment the intact region and “2” denotes the model used to segment the post-breakage region).

Method	Model Size	Number of Pictures	Inference Time	FPS
1	507 M	385	14.371 s	26.79
2	449 M	220	28.740 s	7.64

## Data Availability

Researchers can contact the first author by email to inquire about data availability.
